# Village Forest Experience Program Improves Cognitive Function and Reduces Salivary Cortisol and Oral Pathogens in Older Adults

**DOI:** 10.3390/healthcare14081072

**Published:** 2026-04-17

**Authors:** Mu-Yeol Cho, Je-Hyun Eom, Ji-Won Kim, Yun-Woo Kim, Seung-Jo Yang, Jiyoung Hwang, Mi-Hwa No, Hye-Sung Kim

**Affiliations:** 1 Apple Tree Institute of Biomedical Science, Apple Tree Medical Foundation, Goyang-si 10447, Republic of Korea; 2 DOCSmedi Co., Ltd., Goyang 10387, Republic of Korea

**Keywords:** forest therapy, cognitive function, salivary cortisol, oral pathogenic bacteria, older adults, community-based intervention

## Abstract

**Background/Objectives:** Forest therapy has demonstrated stress-reducing and immune-enhancing effects, yet its simultaneous impact on cognitive function, stress biomarkers, and oral microbiota in older adults remains unexplored. This study aimed to evaluate the effects of an 8-week community-based village forest experience program on cognitive function, salivary cortisol, and oral pathogenic bacteria in community-dwelling older adults. **Methods:** A total of 125 older adults (mean age 82.2 ± 5.3 years; 87.2% female) from 17 senior centers participated in a single-arm, pre–post intervention study. Cognitive function was assessed using the Cognitive Impairment Screening Test (CIST), salivary cortisol was measured by ELISA, and seven oral bacterial species were quantified by qPCR. **Results:** CIST scores improved significantly (*p* = 0.003, d = 0.27), with the suspected cognitive impairment subgroup showing greater improvement (d = 0.66) and 48.8% transitioning to normal classification. Salivary cortisol decreased significantly (*p* = 0.002), and total bacterial load, *Porphyromonas gingivalis*, and *Tannerella forsythia* were significantly reduced. The 80–84-year age group showed the greatest cognitive gain, whereas participants aged 85 and older showed no significant change. **Conclusions:** An accessible village forest program may simultaneously benefit cognitive function, stress, and oral health in older adults with early-stage cognitive decline. Controlled studies are needed to confirm causality and elucidate the underlying mechanisms.

## 1. Introduction

The Republic of Korea is one of the most rapidly aging societies globally, with adults aged 65 years and older constituting 19.2% of the population in 2024 and projected to exceed 30% by 2035 [1]. The pooled prevalence of dementia among Korean older adults has been estimated at 9.2%, with mild cognitive impairment affecting an additional 24.1% [2]. Given the limited efficacy of pharmacological interventions once neurodegeneration has manifested, non-pharmacological community-based approaches targeting early-stage cognitive decline have received increasing attention [3]. Forest therapy, defined as evidence-based health promotion through structured exposure to forest environments, has demonstrated beneficial effects on cortisol reduction, mood regulation, and immune function in systematic reviews [4,5,6].

However, most forest therapy studies targeting older adults have been conducted in designated national healing forests with small sample sizes and have focused primarily on psychological outcomes such as depression and quality of life [7]. Few studies have examined the effectiveness of forest therapy delivered in village forests adjacent to senior centers, which serve as primary social and welfare hubs for community-dwelling older adults and require no dedicated transportation. Evidence on the cognitive and biological effects of forest therapy in this population remains limited.

Recent evidence has established the oral–brain axis as a biologically plausible pathway linking periodontal health to cognitive decline. Periodontal pathogens, particularly *Porphyromonas gingivalis*, have been identified in the brains of patients with Alzheimer’s disease, and their virulence factors have been shown to promote neuroinflammation and tau phosphorylation [8,9]. Chronic psychological stress elevates salivary cortisol, which has been demonstrated to shift the oral microbiome toward a periodontitis-associated transcriptional profile [10] and suppress mucosal immune defenses including secretory immunoglobulin A [11]. These findings indicate a bidirectional relationship among stress, oral pathogens, and cognitive function that forest-based interventions may beneficially modulate through their stress-reducing properties.

Despite this theoretical rationale, no study to date has simultaneously assessed the effects of a forest therapy intervention on cognitive function, stress biomarkers, and oral bacterial profiles within a single cohort. The present study aimed to evaluate the effects of an 8-week community-based village forest experience program on cognitive function as measured by the Cognitive Impairment Screening Test (CIST), salivary cortisol as a stress biomarker, and oral pathogenic bacteria as quantified by quantitative polymerase chain reaction in community-dwelling older adults attending senior centers in the Republic of Korea.

## 2. Materials and Methods

### 2.1. Study Design and Ethical Approval

This study employed a single-arm, pre–post intervention design to evaluate the effects of a community-based village forest experience program on cognitive function, stress biomarkers, and oral bacterial profiles in community-dwelling older adults. The study protocol was approved by the Apple Tree Dental Hospital Institutional Review Board (IRB No. ATDH-2025-0003; approved on 4 February 2025) and registered with the Clinical Research Information Service (CRIS: KCT0011498). All participants provided written informed consent prior to enrollment, including consent for the collection, storage, and analysis of biological specimens. The study was conducted in accordance with the Declaration of Helsinki. No adverse events were reported during the program.

### 2.2. Participants

Participants were recruited from senior centers in Goyang-si, Gyeonggi Province, Republic of Korea, between March and June 2025. The program was part of a community forest welfare initiative supported by the Korea Forest Welfare Institute for dementia prevention through the Green Fund.

Eligible participants were aged 65 years or older, regularly attended a participating senior center, and were able to walk independently or with minimal assistance to nearby village forests. Participants were excluded if they had recently used antibiotics, were concurrently enrolled in another clinical study, attended fewer than two program sessions, or consumed food or beverages immediately prior to sample collection. Only participants with complete pre- and post-intervention data (CIST scores, cortisol values, and total bacteria Ct values at both time points) were included in the final analysis.

**Figure 1:**
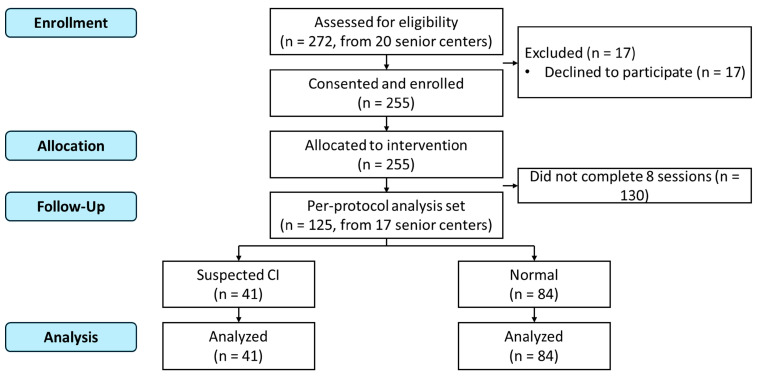
Participant flow diagram.

A total of 272 individuals from 20 senior centers were screened, of whom 255 provided written informed consent. After applying the above criteria, 125 participants from 17 senior centers constituted the final analytic sample (Figure 1).

### 2.3. Intervention

The village forest experience program consisted of eight weekly sessions, each lasting two hours, conducted in village forests within walking distance of each participating senior center. Each session was led by a certified forest therapy instructor and comprised three phases: (1) a warm-up phase (30 min) including stretching and sensory awakening activities; (2) a forest activity phase (60 min) consisting of forest trail walking, barefoot walking, guided forest interpretation, and forest meditation; and (3) a group dialog phase (30 min) featuring facilitated peer conversations, tea, and group singing. When inclement weather prevented outdoor activities, sessions were adapted for indoor spaces. Data collection was performed at the beginning of Session 1 (baseline) and Session 8 (post-intervention), prior to the commencement of any program activity.

### 2.4. Outcome Measures

#### 2.4.1. Cognitive Function

Cognitive function was assessed using the Cognitive Impairment Screening Test (CIST), a nationally standardized instrument for community-based cognitive screening developed by the National Institute of Dementia in the Republic of Korea [12]. The CIST evaluates orientation, memory, attention, visuospatial function, executive function, and language, yielding a total score of 0–30. Age- and education-adjusted cutoff scores are used to classify individuals as “Normal” or “Suspected cognitive impairment (CI).” The CIST was administered by trained evaluators at both time points.

#### 2.4.2. Salivary Cortisol

Unstimulated whole saliva samples were collected into sterile conical tubes. Participants were instructed to refrain from eating, drinking (except water), and smoking for at least 30 min prior to collection. Samples were collected between 10:00 a.m. and 2:00 p.m., with collection times kept consistent within each center across both time points. Samples were transported on ice, centrifuged, and stored at −20 °C until analysis. Salivary cortisol concentrations (μg/dL) were determined by enzyme-linked immunosorbent assay (ELISA) at EONE Laboratories (Incheon, Republic of Korea).

#### 2.4.3. Oral Bacteria

Oral microbial samples were collected using the Oralbiome CHECK kit (Docsmedi Co., Ltd., Goyang, Republic of Korea), which included gargle solution and dedicated collection tubes. Participants gargled vigorously for 30 s with the provided solution and then expectorated into the collection tube. Genomic DNA was extracted using the Bacteria Genomic DNA Isolation Kit (LaboPass, COSMO Genetech, Seoul, Republic of Korea) according to the manufacturer’s instructions. Quantitative real-time polymerase chain reaction (qPCR) was performed by DOCSMEDI (Seoul, Republic of Korea) to determine cycle threshold (Ct) values for total bacteria and six species of periodontal and cariogenic pathogens: *Porphyromonas gingivalis* (Pg), *Treponema denticola* (Td), *Tannerella forsythia* (Tf), *Prevotella intermedia* (Pi), *Campylobacter rectus* (Cr), *Fusobacterium nucleatum* (Fn), and *Streptococcus mutans* (Sm). Higher Ct values correspond to lower bacterial DNA concentrations; thus, an increase in Ct from pre- to post-intervention indicates a reduction in bacterial load. Samples with no amplification signal were excluded from species-specific analyses, resulting in variable sample sizes across species.

### 2.5. Statistical Analysis

Pre–post changes were analyzed using paired *t*-tests, and effect sizes were calculated as Cohen’s *d*. Changes in cognitive classification (Normal vs. Suspected CI) were evaluated using the McNemar test. Subgroup analyses were performed by baseline cognitive status, sex, and age group. All analyses were performed using R version 4.3.1 (R Foundation for Statistical Computing, Vienna, Austria), with a two-tailed *p*-value < 0.05 considered statistically significant. No adjustments for multiple comparisons were applied, as the analyses were exploratory. A formal sample size calculation was not performed a priori, as this was an exploratory study. The sample size was determined by the number of eligible participants from the 20 senior centers participating in the community forest welfare program.

## 3. Results

### 3.1. Participant Characteristics

The demographic and baseline characteristics of the 125 participants are presented in Table 1. The mean age was 82.2 ± 5.3 years (range: 68–97), with 109 females (87.2%) and 16 males (12.8%). The largest age group was 80–84 years (35.2%), followed by ≥85 years (32.8%). Mean years of education were 8.9 ± 4.4. At baseline, 84 participants (67.2%) were classified as “Normal” and 41 (32.8%) as “Suspected cognitive impairment” based on age- and education-adjusted CIST cutoffs.

### 3.2. Changes in Cognitive Function

Overall CIST scores improved significantly from 21.00 ± 6.33 to 22.22 ± 6.03 (+1.22, *p* = 0.003; Table 2). The improvement was driven by the suspected CI subgroup, which showed a mean increase of 3.44 points (*p* < 0.001, d = 0.66), whereas the normal subgroup showed no significant change (*p* = 0.749). By sex, significant improvement was observed among females (*p* = 0.002) but not males (*p* = 0.755). By age group, participants aged 80–84 years showed the most pronounced improvement (*p* = 0.009), while no significant changes were observed in the other age groups (Table 3).

Regarding cognitive classification changes (Table 4), 20 of 41 participants (48.8%) initially classified as suspected CI transitioned to normal after the intervention, while only three of 84 (3.6%) in the normal group declined to suspected CI. The McNemar test confirmed this asymmetry (χ^2^ = 11.13, *p* < 0.001).

### 3.3. Changes in Salivary Cortisol

Salivary cortisol decreased significantly from 0.12 ± 0.07 to 0.10 ± 0.06 μg/dL (*p* = 0.002; Table 2). When stratified by cognitive status (Table 5), the reduction was significant in the normal group (*p* = 0.004) but not in the suspected CI group (*p* = 0.173).

### 3.4. Changes in Oral Bacteria

Significant increases in Ct values—reflecting reduced bacterial DNA concentrations, as higher Ct values correspond to lower bacterial loads—were observed for total bacteria (*p* = 0.026), *P. gingivalis* (*p* = 0.034), and *T. forsythia* (*p* = 0.003; Table 2). No significant changes were observed for the remaining species. When stratified by baseline cognitive status (Table 5), *T. forsythia* showed a notably larger reduction in the suspected CI group (+1.16, *p* = 0.006, d = 0.50) compared to the normal group (*p* = 0.083).

## 4. Discussion

This study simultaneously evaluated the effects of a community-based village forest experience program on cognitive function, salivary cortisol, and oral bacteria in a single cohort of older adults. Most previous forest therapy studies have assessed psychological indicators or stress biomarkers in isolation, and no study has incorporated a multidimensional assessment encompassing cognitive function and oral microbiota. Following the 8-week program, significant improvements in CIST scores, reductions in salivary cortisol, and decreases in key periodontal pathogens were observed.

The largest effect was observed in the suspected cognitive impairment (CI) group. Although CIST scores improved significantly across all participants, the suspected CI subgroup showed a mean increase more than twice that of the overall sample, with 48.8% transitioning to normal classification, whereas the normal group showed no significant change. This suggests that forest-based interventions may be associated with greater benefit in older adults at the early stages of cognitive decline. The observed associations should not be attributed solely to forest exposure per se, as the intervention was inherently multi-component in nature. Physical activity through forest trail walking and barefoot walking, cognitive stimulation through identifying and recalling the names of trees and plants, social interaction through peer dialog, and sensory engagement through guided forest meditation each represent potentially active components that may have independently or synergistically contributed to the observed outcomes. Previous studies have reported that physical activity in natural environments promotes cerebral blood flow and neuroplasticity [13,14], and evidence supports the role of social engagement and cognitive stimulation in maintaining cognitive reserve [15,16]. Park et al. (2024) administered a 20-week standardized anti-aging forest healing program to 33 older adults aged 70 years and older, recruited from dementia relief centers, and reported significant improvements in CIST total scores [17]. The present study confirmed comparable cognitive improvements with a shorter 8-week program, in everyday village forests accessible on foot from senior centers rather than in designated national healing forests, and in a sample of 125 participants. It should be noted, however, that the present study was not powered for subgroup analyses; these findings are therefore exploratory and should be interpreted with caution.

Significant improvement was observed in females but not in males; however, the male subsample comprised only 16 participants, limiting statistical power for this comparison. By age group, the greatest cognitive improvement was observed in the 80–84-year group. Korean dementia epidemiological data indicate that the prevalence of cognitive impairment and dementia increases from age 75, with age-specific prevalence doubling every 5–6 years [2,18]. In the present study, the proportion of suspected CI also increased with age; however, the 85-years-and-older group, which had the highest proportion of suspected CI, showed no significant improvement. This pattern suggests that participants aged 80–84 years may be at a stage where cognitive decline has begun but remains responsive to intervention. The absence of significant improvement in the ≥85 group may reflect a combination of factors beyond cognitive decline alone, including greater physical frailty [19] and reduced neuroplasticity associated with advanced age [20], both of which may have limited the effectiveness of an 8-week program in producing measurable cognitive change. Given the small sample sizes within individual age subgroups and the exploratory nature of these analyses, these age-related patterns warrant confirmation in adequately powered future studies.

The significant reduction in salivary cortisol is consistent with existing evidence for the stress-reducing effects of forest environments [4,5]. It should be noted that saliva samples were collected immediately before the start of each data collection session (Session 1 and Session 8), prior to any physical activity. The observed reduction in salivary cortisol therefore may reflect a change in resting-state cortisol levels following seven weeks of program participation, rather than an acute exercise-induced response. The cortisol reduction was significant only in the normal cognition group and not in the suspected CI group. One possible explanation for the absence of a significant cortisol reduction in the suspected CI subgroup is HPA axis dysregulation associated with early cognitive decline. Ouanes and Popp (2019) reviewed evidence linking elevated cortisol levels and altered HPA axis reactivity to increased risk of dementia [21]. Johar et al. (2015) further reported that older adults with cognitive impairment exhibited blunted diurnal cortisol patterns, characterized by lower morning and higher evening cortisol levels, compared to cognitively healthy individuals [22]. These findings suggest that HPA axis reactivity may be altered in the context of cognitive decline, which could attenuate the cortisol response to stress-reducing stimuli such as the present intervention.

The significant reductions in total bacterial load, *P. gingivalis*, and *T. forsythia* are, to our knowledge, the first such findings reported in a forest therapy study. These are pathogenic bacteria strongly associated with periodontal disease [23]. *T. forsythia* showed a larger reduction in the suspected CI group (d = 0.50) than in the normal group (d = 0.20), and this pattern occurred in parallel with the cognitive improvement observed in the same subgroup. Recent studies have proposed the oral–brain axis hypothesis, whereby periodontal pathogens reach the brain via the bloodstream and promote neuroinflammation [8,9]. The present study did not directly test this pathway; however, the concurrent reductions in cortisol and periodontal pathogens raise the possibility that stress reduction may have improved oral immune function, potentially contributing to decreased pathogenic bacterial load. These findings should be interpreted with caution, however, as data on participants’ oral hygiene practices, dietary habits, and dental care were not collected and may represent important unmeasured confounders. Future controlled studies incorporating mediation analysis are needed to directly examine the validity and directionality of this proposed pathway.

This study has several limitations. The single-arm design without a control group precludes causal inference, as the observed changes may reflect regression to the mean, practice effects on cognitive testing, or seasonal variations. Although saliva collection times were kept consistent within each center across both time points, collection times varied across centers. Given the well-established diurnal pattern of cortisol secretion, this inter-center variation may have introduced systematic differences in absolute cortisol levels between centers, representing a potential source of confounding that should be considered when interpreting the cortisol findings. The predominantly female sample (87.2%) limits the generalizability of sex-specific findings. The absence of data on participants’ concurrent activities, medications, and health behaviors represents potential unmeasured confounders. Adherence data were not collected, and the potential influence of variation in individual session attendance on the observed outcomes could not be assessed. Future studies should incorporate systematic attendance tracking to allow for dose–response analyses. Attrition from 272 to 125 participants, while typical in community-based elderly research, may have introduced selection bias. A comparison of available baseline characteristics (age, sex, and baseline CIST scores) between the final analytic sample and excluded participants revealed no significant differences (all *p* > 0.05), suggesting that attrition was unlikely to have introduced systematic selection bias on these measures. No a priori sample size calculation was performed, as this was an exploratory study with a sample size determined by program enrollment. The statistical power available for subgroup analyses was therefore limited, and subgroup findings should be interpreted accordingly. Additionally, CIST evaluators were not blinded to the assessment time point, which may have introduced measurement bias. Despite these limitations, this study is the first to explore the multidimensional health effects of forest therapy by simultaneously measuring cognitive function, stress biomarkers, and oral microbiota in a single cohort. The multi-site data from 125 participants across 17 senior centers, obtained in village forests accessible without dedicated transportation, provide initial evidence for the integration of community-based forest programs into dementia prevention strategies.

## 5. Conclusions

An 8-week village forest experience program was associated with improved cognitive function, reduced salivary cortisol, and decreased periodontal pathogenic bacteria in community-dwelling older adults. The greatest cognitive benefit was observed in the suspected cognitive impairment subgroup. As a single-arm exploratory study, these results require confirmation through randomized controlled trials with extended follow-up and mediation analyses to elucidate the mechanisms underlying these multidimensional effects.

## Figures and Tables

**Table 1:** Demographic and baseline characteristics of participants (N = 125).

Characteristic	n or Mean ± SD	% or Range
Sex		
Female	109	87.2
Male	16	12.8
Age (years)	82.2 ± 5.3	68–97
Age group		
≤74	8	6.4
75–79	32	25.6
80–84	44	35.2
≥85	41	32.8
Education (years)	8.9 ± 4.4	0–18
Education level		
No formal education/1–5 years	13	10.4
Elementary school (6 years)	29	23.2
Middle school (7–9 years)	30	24.0
High school (10–12 years)	38	30.4
College or above (≥13 years)	15	12.0
Number of senior centers	17	
Baseline cognitive status (CIST) ^a^		
Normal	84	67.2
Suspected cognitive impairment	41	32.8
Suspected cognitive impairment by age group ^b^		
≤74	2	25
75–79	6	18.8
80–84	14	31.8
≥85	20	48.8

^a^ Cognitive status was classified based on the Korean version of the Cognitive Impairment Screening Test (CIST) using age- and education-adjusted cutoff scores. ^b^ Values represent the number and proportion of participants classified as suspected cognitive impairment within each age group. Values are presented as n (%) for categorical variables and mean ± SD (range) for continuous variables. SD, standard deviation; CIST, Cognitive Impairment Screening Test.

**Table 2:** Pre- and post-intervention comparison of outcome variables.

Variable	n	Pre (Mean ± SD)	Post (Mean ± SD)	*p*-Value	Cohen’s *d*
Cognitive function					
CIST score (total)	125	21.00 ± 6.33	22.22 ± 6.03	0.003	+0.27
Suspected CI subgroup	41	13.95 ± 4.12	17.39 ± 5.11	<0.001	+0.66
Normal subgroup	84	24.44 ± 3.90	24.57 ± 4.96	0.749	+0.04
Stress biomarker					
Salivary cortisol (μg/dL)	125	0.12 ± 0.07	0.10 ± 0.06	0.002	−0.29
Oral bacteria (Ct value)					
Total bacteria	125	22.61 ± 1.83	23.04 ± 1.73	0.026	+0.20
*P. gingivalis*	101	26.58 ± 2.88	27.06 ± 2.82	0.034	+0.21
*T. denticola*	87	29.49 ± 3.35	30.10 ± 2.86	0.070	+0.20
*T. forsythia*	110	29.05 ± 2.96	29.77 ± 3.02	0.003	+0.29
*P. intermedia*	74	28.93 ± 3.39	29.14 ± 3.21	0.454	+0.09
*C. rectus*	44	30.29 ± 3.51	31.01 ± 2.84	0.112	+0.24
*F. nucleatum*	125	27.08 ± 3.09	27.35 ± 2.84	0.281	+0.10
*S. mutans*	51	35.11 ± 2.65	34.98 ± 2.71	0.770	−0.04

CIST, Cognitive Impairment Screening Test; CI, cognitive impairment; Ct, cycle threshold. Higher Ct values indicate lower bacterial DNA concentrations; thus, an increase in Ct from pre- to post-intervention reflects a reduction in bacterial load.

**Table 3:** Changes in CIST scores by sex and age group.

Variable	n	Pre (Mean ± SD)	Post (Mean ± SD)	*p*-Value	Cohen’s *d*
Sex					
Female	109	20.93 ± 6.52	22.36 ± 6.20	0.002	+0.31
Male	16	21.50 ± 5.02	21.25 ± 4.77	0.755	−0.08
Age group (years)					
≤74	8	24.75 ± 3.65	26.88 ± 2.53	0.013	+1.18
75–79	32	24.72 ± 5.34	25.66 ± 4.12	0.150	+0.26
80–84	44	20.52 ± 5.86	22.36 ± 5.63	0.004	+0.45
≥85	41	17.88 ± 6.23	18.46 ± 6.00	0.524	+0.10

CIST, Cognitive Impairment Screening Test.

**Table 4:** Changes in cognitive classification before and after intervention.

Pre → Post Classification	n	% of Pre Subgroup	McNemar Test
Suspected CI group (n = 41)			χ^2^ = 11.130, *p* <0.001
Improved (Suspected CI → Normal)	20	48.8	
Unchanged (Suspected CI → Suspected CI)	21	51.2	
Normal group (n = 84)			
Worsened (Normal → Suspected CI)	3	3.6	
Unchanged (Normal → Normal)	81	96.4	

Cognitive status was classified based on age- and education-adjusted CIST cutoff scores. McNemar test was performed with continuity correction. CI, cognitive impairment; CIST, Cognitive Impairment Screening Test.

**Table 5:** Pre- and post-intervention outcomes by baseline cognitive status.

Variable	n	Pre (Mean ± SD)	Post (Mean ± SD)	*p*-Value	Cohen’s *d*
Salivary cortisol (μg/dL)					
Suspected CI	41	0.13 ± 0.07	0.12 ± 0.06	0.173	−0.22
Normal	84	0.12 ± 0.07	0.09 ± 0.05	0.004	−0.32
Oral bacteria (Ct value)					
Total bacteria					
Suspected CI	41	22.52 ± 1.58	23.03 ± 1.81	0.107	+0.26
Normal	84	22.66 ± 1.95	23.04 ± 1.69	0.109	+0.18
*P. gingivalis*					
Suspected CI	32	25.84 ± 2.83	26.62 ± 2.73	0.067	+0.34
Normal	69	26.92 ± 2.85	27.26 ± 2.86	0.207	+0.15
*T. denticola*					
Suspected CI	26	30.07 ± 3.70	30.67 ± 3.27	0.340	+0.19
Normal	61	29.25 ± 3.20	29.86 ± 2.66	0.128	+0.20
*T. forsythia*					
Suspected CI	35	28.65 ± 3.08	29.81 ± 3.11	0.006	+0.50
Normal	75	29.24 ± 2.91	29.75 ± 2.99	0.083	+0.20
*P. intermedia*					
Suspected CI	22	28.61 ± 3.87	29.02 ± 3.44	0.466	+0.16
Normal	52	29.06 ± 3.19	29.18 ± 3.15	0.702	+0.05
*C. rectus*					
Suspected CI	15	29.96 ± 2.83	29.99 ± 2.88	0.967	+0.01
Normal	29	30.46 ± 3.85	31.54 ± 2.72	0.070	+0.35
*F. nucleatum*					
Suspected CI	41	27.17 ± 3.05	27.37 ± 2.87	0.579	+0.09
Normal	84	27.04 ± 3.13	27.34 ± 2.84	0.358	+0.10
*S. mutans*					
Suspected CI	19	34.31 ± 2.30	34.85 ± 2.33	0.437	+0.18
Normal	32	35.58 ± 2.76	35.05 ± 2.94	0.358	−0.16

Values are presented as mean ± SD. n varies across bacterial species due to “undetermined” qPCR results. CI, cognitive impairment; Ct, cycle threshold; qPCR, quantitative polymerase chain reaction.

## Data Availability

The data presented in this study are available on request from the corresponding author. The data are not publicly available due to ethical restrictions, as the dataset contains sensitive health information of elderly participants, including cognitive assessment scores, salivary cortisol levels, and oral microbial profiles, collected under IRB approval with informed consent specifying that individual-level data would remain confidential.

## Abbreviations

The following abbreviations are used in this manuscript:

CIST	Cognitive Impairment Screening Test
CI	Cognitive Impairment
Ct	Cycle Threshold
ELISA	Enzyme-Linked Immunosorbent Assay
qPCR	Quantitative Polymerase Chain Reaction
IRB	Institutional Review Board
CRIS	Clinical Research Information Service
